# Role of *Drosophila* Amyloid Precursor Protein in Memory Formation

**DOI:** 10.3389/fnmol.2016.00142

**Published:** 2016-12-08

**Authors:** Thomas Preat, Valérie Goguel

**Affiliations:** Genes and Dynamics of Memory Systems, Brain Plasticity Unit, Centre National de la Recherche Scientifique (CNRS), ESPCI Paris, PSL Research UniversityParis, France

**Keywords:** *Drosophila melanogaster*, amyloid precursor protein, learning, memory, mushroom bodies, conditional expression, amyloid peptide

## Abstract

The amyloid precursor protein (APP) is a membrane protein engaged in complex proteolytic pathways. APP and its derivatives have been shown to play a central role in Alzheimer’s disease (AD), a progressive neurodegenerative disease characterized by memory decline. Despite a huge effort from the research community, the primary cause of AD remains unclear, making it crucial to better understand the physiological role of the APP pathway in brain plasticity and memory. *Drosophila melanogaster* is a model system well-suited to address this issue. Although relatively simple, the fly brain is highly organized, sustains several forms of learning and memory, and drives numerous complex behaviors. Importantly, molecules and mechanisms underlying memory processes are conserved from flies to mammals. The fly encodes a single non-essential APP homolog named APP-Like (APPL). Using *in vivo* inducible RNA interference strategies, it was shown that APPL knockdown in the mushroom bodies (MB)—the central integrative brain structure for olfactory memory—results in loss of memory. Several APPL derivatives, such as secreted and full-length membrane APPL, may play different roles in distinct types of memory phases. Furthermore, overexpression of *Drosophila* amyloid peptide exacerbates the memory deficit caused by APPL knockdown, thus potentiating memory decline. Data obtained in the fly support the hypothesis that APP acts as a transmembrane receptor, and that disruption of its normal function may contribute to cognitive impairment during early AD.

## *Drosophila* as a Model to Study the Role of the APP Pathway in Memory

The initial events leading to Alzheimer’s disease (AD) are still unknown. Amyloid deposits, a hallmark of AD, are formed by the aggregation of amyloid peptides (Aβ) resulting from proteolytic processing of the amyloid precursor protein (APP; Turner et al., 2003). APP is a transmembrane protein that is subjected to two exclusive proteolytic pathways: the non-amyloidogenic pathway initiated by the α-secretase producing a secreted APP form (sAPPα), and the amyloidogenic pathway initiated by the β-secretase leading to the production of Aβ. For many years, the amyloid hypothesis put Aβ at the center of the etiology of AD (Hardy and Selkoe, 2002). However, several studies have shown that APP plays a positive role in memory (Meziane et al., 1998; Ring et al., 2007), raising the possibility that aside from Aβ toxicity, APP loss-of-function may participate in AD, particularly during the early stages of the disease characterized by memory impairment. The physiological role of APP is difficult to assess due to its numerous proteolytic metabolites. Functional studies of the APP pathway in rodents are also limited due to the redundancy of the three APP-related genes and the lethality of the triple knockout (Heber et al., 2000; Herms et al., 2004). In addition, mouse studies have been essentially performed using constitutive mutants, making it hard to discriminate developmental functions from direct roles in the adult brain. The *Drosophila melanogaster* genome contains homologs of 75% of human disease-related genes (Fortini et al., 2000; Reiter et al., 2001). Interestingly, the fly expresses a single non-essential APP ortholog, called APP-Like (APPL). APPL is a neuronal-specific protein particularly expressed in the axonal neuropil of the adult mushroom bodies (MB; Torroja et al., 1996).

*Appl-*deficient flies (*Appl^d^*) display phototaxis deficits that are alleviated upon ectopic expression of human APP (hAPP), which is the first demonstration that APPL is an APP ortholog (Luo et al., 1992). APPL/hAPP sequence comparison found homology regions at the E1 and E2 ectodomains and at the C-terminal intracellular domain (Rosen et al., 1989). APPL protein (887 aa) is substantially longer than hAPP (695 aa), largely due to having longer sequences between E1 and E2 domains and between E2 and Aβ sequences. Aβ sequences are manifestly not conserved between APP and APPL, and amyloid peptides are not described in wild-type *Drosophila*. However, a *Drosophila* Aβ-like peptide (dAβ) was identified in old flies overexpressing APPL (Carmine-Simmen et al., 2009). Indeed, APPL overexpression in old age leads to Thioflavin-S-positive aggregates that are associated with neurodegeneration, suggesting that APPL processing produces an analog of human Aβ (Carmine-Simmen et al., 2009). Importantly, APPL undergoes similar proteolytic pathways to APP (Poeck et al., 2011), and the homologs of all mammalian secretases have been characterized in the fly (Rooke et al., 1996; Boulianne et al., 1997; Hong and Koo, 1997; Carmine-Simmen et al., 2009).

Despite its relative simplicity, the fly brain is highly structured and drives sophisticated behaviors. In particular, it is extensively used as a model system to study associative memory. Molecular mechanisms underlying memory are conserved from flies to mammals (McGuire et al., 2005), and the neuronal structures involved are well described (Heisenberg, 2003; Waddell, 2010; Aso et al., 2014a,b). The MB are known as the central integrative brain structure for olfactory associative memory (de Belle and Heisenberg, 1994; Pascual and Préat, 2001; Gerber et al., 2004; Krashes et al., 2007; Gervasi et al., 2010). The MB are a bilateral structure composed of 4000 intrinsic neurons, the Kenyon cells, classed into three subtypes whose axons form two vertical (α and α’) and three medial (β, β’ and γ) lobes (Crittenden et al., 1998). Using a classical conditioning paradigm in which an odorant is paired with the delivery of electric shocks, the fly is capable of forming six discrete aversive memory phases reflected at neural network level (Bouzaiane et al., 2015). Learning and short-term memory are measured immediately after a single conditioning, while middle-term memory (MTM) is assessed 1–3 h later. The fly can also produce two antagonistic forms of consolidated memory (Isabel et al., 2004). Long-term anesthesia-resistant memory (LT-ARM) is formed after multiple massed cycles of conditioning, whereas the robust long-term memory (LTM) is only formed after multiple cycles spaced by rest intervals. Crucially, LTM is the only memory phase dependent on *de novo* protein-synthesis (Tully et al., 1994).

Many human neurodegenerative diseases can be modeled in *Drosophila* (Bilen and Bonini, 2005). In particular, transgenic flies have been generated to analyze human Aβ-induced toxicity. Expression in the *Drosophila* brain of human Aβ42 resulted in defects similar to that observed in the mouse (Finelli et al., 2004; Greeve et al., 2004; Iijima et al., 2004, 2008; Crowther et al., 2005; Zhao et al., 2010). Thus, similarities between Aβ-induced neurotoxic biochemical pathways in flies and humans make *Drosophila* a relevant model to study the molecular basis of AD pathogenesis. Neuronal expression of human Aβ42 leads to a learning deficit in young flies, and MTM deficit in older flies (Iijima et al., 2004, 2008; Fang et al., 2012). Likewise, neuronal overexpression of hAPP alters learning and MTM in young flies and these deficits become more pronounced as the fly ages (Sarantseva et al., 2009). hAPP expression in the MB was also shown to alter LTM (Goguel et al., 2011).

APP overexpression-related memory deficits likely result from accumulation of amyloid peptides, especially as it was suggested that the fly secretases can cleave APP (Greeve et al., 2004). Furthermore, the above-cited results were obtained using constitutive overexpression, creating conditions under which APP and/or Aβ accumulate over the entire life of the fly, thus increasing their toxic potential, particularly during developmental stages. In fact, when dAβ overexpression is achieved in the MB of adult flies for only 2 days, no MTM deficit is observed (Bourdet et al., 2015a). Taken together, the data suggest that memory impairments observed with constitutive expression of APP result from a developmental defect and/or general neuronal dysfunction rather than from some specific alteration of the molecular mechanisms required to sustain memory formation.

## APPL Is Required for Specific Memory Phases

Aβ toxicity has been a focus of research for years, but it now appears essential to better understand APP function in brain physiology. Early on, it was shown that *Appl^d^* flies do not form normal associative learning, but it was impossible to conclude that APPL was involved in this process as the *Appl^d^* flies did not react normally to electric shock exposure, which was the unconditioned stimulus used for the study (Luo et al., 1992). It was later shown that *Appl* disruption leads to slight abnormalities in the morphology of the MB lobes (Li et al., 2004). More recently, a study demonstrated the role of APPL in brain wiring (Soldano et al., 2013). Thus, functional studies need to rule out possible roles during brain development. One of the major advantages of the *Drosophila* model is that it can be used to implement inducible loss-of-function studies. Indeed, the expression of any gene of interest can be controlled both spatially (Brand and Perrimon, 1993) and temporally (McGuire et al., 2003).

Using conditional RNA interference, it was demonstrated that APPL expression in the adult MB is required for the proper formation of specific memory phases. APPL silencing in the MB of adult flies was shown to disrupt MTM and LTM, but neither learning nor ARM formation was affected (Goguel et al., 2011; Bourdet et al., 2015b). MTM and LTM are two memory phases known to share identical neuronal circuits (Bouzaiane et al., 2015), indicating a role for APPL in these structures. These memory phenotypes are reminiscent of the pattern displayed by *amnesiac* mutants (Quinn et al., 1979; Feany and Quinn, 1995; DeZazzo et al., 1999; Yu et al., 2006), suggesting that APPL and Amnesiac, a predicted neuropeptide precursor showing homology to an adenylate cyclase-activating peptide (Feany and Quinn, 1995), are involved in the same molecular pathways.

Memory deficits are thus caused by loss of APPL function, independent of the amyloid pathway toxicity. This data further supports the hypothesis that APP downregulation might contribute to early cognitive impairment in AD. To further assess which APPL fragment is required for memory processes, two APPL-mutant forms were used: a constitutively-secreted APPL protein (APPL^s^) and a non-cleavable secretion-defective form (APPL^sd^). The *Appl^s^* sequence contains a stop codon that generates a soluble 788-amino-acid N-terminal fragment of APPL, whereas APPL^sd^ is deleted from the α and β cleavage sites, thus preventing its processing (Torroja et al., 1996, 1999). Consequently, APPL^sd^ is exclusively expressed as a transmembrane protein. Overexpression of APPL^s^ in the adult MB rescued the MTM deficit caused by a reduction of endogenous APPL levels, indicating that a secreted fragment of APPL is involved in memory (Bourdet et al., 2015b). This is consistent with mammalian studies showing a role for sAPPα in memory (Meziane et al., 1998; Bour et al., 2004; Ring et al., 2007; Taylor et al., 2008). Unexpectedly, however, overexpression of the fly α-secretase KUZ (Rooke et al., 1996), thought to increase sAPPLα levels, did not rescue the memory deficit caused by APPL partial loss-of-function, and even further exacerbated the MTM impairment (Bourdet et al., 2015b). Interestingly, KUZ overexpression in this context was shown to decrease full-length APPL (fl-APPL) protein levels, prompting the hypothesis that the exacerbation of the memory phenotype resulted from a reduction of fl-APPL levels. Supporting this hypothesis, transient APPL^sd^ expression in the MB was also able to restore wild-type MTM in an APPL knockdown background (Bourdet et al., 2015b). Interestingly, neither APPL^s^ nor APPL^sd^ overexpression rescued the LTM phenotype of APPL partial loss-of-function flies. Although negative results are difficult to interpret, they may indicate distinct molecular APPL requirements for MTM and LTM.

Taken together, the data indicate that both fl-APPL and sAPPL are involved in MTM. This apparently contradicts a previous study showing that sAPPα could rescue the spatial learning defect of APP knockout mice (Ring et al., 2007). However, the APPL proteins APLP1 and APLP2 were preserved in that study. As the three APP homologs show some functional redundancy (Anliker and Müller, 2006), disruption of full-length APP functions might have been partially fulfilled by APLP1 or APLP2. Memory function cannot therefore be attributed exclusively to sAPPα.

Interestingly, APP may be a receptor for sAPPα (Young-Pearse et al., 2008; Gralle et al., 2009). In *Drosophila*, sAPPL was shown to act as a soluble ligand for neuroprotective functions (Wentzell et al., 2012). Moreover, co-immunoprecipitation experiments from transfected Kc cells uncovered an interaction between fl-APPL and sAPPL, suggesting that sAPPL could be a ligand for fl-APPL (Wentzell et al., 2012). It is thus tempting to speculate that APPL is involved in MTM processes through a sAPPL/fl-APPL ligand/receptor interaction.

## Aβ Exacerbates the Memory Deficit Caused by APPL Partial Loss-of-Function in *Drosophila*

Neurotoxic effects of Aβ accumulation have been well documented, and studies have shown that the β-APP cleavage enzyme, Beta-secretase 1 (BACE1), has a negative impact on memory. In mice models of AD, BACE1 deficiency rescues memory deficits (Ohno et al., 2004, 2007), and conversely, expression of hBACE1 was shown to worsen learning and memory deficits (Rockenstein et al., 2005; Chen et al., 2012). In normal mice, hBACE1 gene knock-in caused AD-relevant cognitive impairment (Plucińska et al., 2014). The authors concluded that low hBACE1 levels were sufficient to cause the formation of toxic Aβ oligomeric assemblies. It is important to note here that hBACE1 knock-in mouse also generated decreased full-length APP levels (Plucińska et al., 2014), a feature that could participate in cognitive impairment. In *Drosophila*, overexpression of the fly β-secretase (dBACE; Carmine-Simmen et al., 2009) in the adult MB did not impact MTM (Bourdet et al., 2015a). In contrast, it exacerbated the memory deficit of low-APPL-level flies (Bourdet et al., 2015a). One possibility is that, similar to KUZ overexpression, an increase of dBACE-mediated processing reduces fl-APPL levels, thus aggravating the memory deficit caused by APPL knockdown. Interestingly, similar results were observed with dAβ: dAβ expression in adult MB neurons impaired MTM only in an APPL partial loss-of-function background (Bourdet et al., 2015a). It was hypothesized that both dBACE and dAβ expression exacerbate the memory deficit caused by a reduction of APPL levels through similar mechanisms mediated by an increase in dAβ production that has knock-on effects on APPL function. Memory would thus be affected by two related processes—APPL downregulation and Aβ toxicity—uncovering a functional link between APPL and Aβ.

## A Physiological Role for Aβ in Memory?

Several reports have shown that at very low physiological concentrations, Aβ modulates synaptic strength (Kamenetz et al., 2003; Abramov et al., 2009) and enhances memory (Puzzo et al., 2008, 2012; Garcia-Osta and Alberini, 2009; Morley et al., 2010). Aβ appears to be a modulator of synaptic activity requiring a fine balance between production and removal. Neprilysins are the major Aβ-degrading enzymes (Iwata et al., 2001) and, as such, are thought to be key to AD. Neprilysin proteins are zinc-dependent endopeptidases known to inactivate small peptides. Their active site faces the extracellular space, and they can be present at presynaptic sites (Fukami et al., 2002; Iwata et al., 2004). Neprilysins play a major role in brain function by terminating neuropeptide signaling at the cell surface, and they are involved in many neuronal processes from axonal regeneration and synaptic plasticity to neuro-inflammation, while at the behavioral level neprilysins have been implicated in motor function, anxiety, circadian rhythms and sleep (Nalivaeva et al., 2012).

The issue of whether neprilysins are involved in memory in non-pathological conditions has been addressed in *Drosophila*. Four neprilysins are expressed in adult *Drosophila* brain (Meyer et al., 2011), and we have shown using inducible RNA interference that they are all required for MTM and LTM (Turrel et al., 2016). We have proposed that these neprilysins target several neuropeptides involved in memory processes (Turrel et al., 2016). An attractive hypothesis is that one of these targets might be Aβ peptide derived from physiological APPL processing. Consistently, the memory phenotypes observed after neprilysin silencing are reminiscent of the specific pattern in APPL mutants: only MTM and LTM are impaired, suggesting a functional interaction between neprilysins and APPL. Neprilysin 2 would be a good candidate here, since several studies have shown that it is capable of degrading human Aβ42 (Finelli et al., 2004; Cao et al., 2008).

## Conclusions

**Figure 1:**
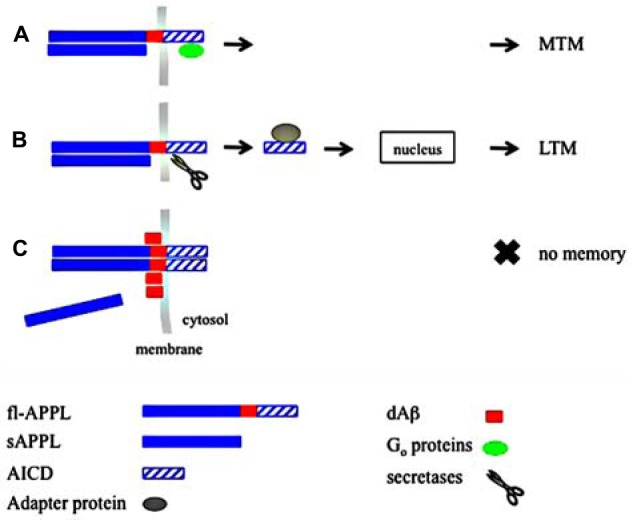
**Model for amyloid precursor protein-Like (APPL) function in memory. (A)** Secreted APPL (sAPPL) and full-length APPL (fl-APPL) interact to induce middle-term memory (MTM) formation via a signaling pathway such as G protein activation. **(B)** After sAPPL and fl-APPL have interacted, secretases produce APPL intracellular domain (AICD). After translocation into the nucleus, AICD activates the transcription required for long-term memory (LTM) formation. **(C)** dAβ inhibits APPL: *Drosophila* Aβ-like peptide (dAβ) binding to APPL promotes APPL cis-dimerization thus preventing sAPPL/fl-APPL interaction.

In the fly, both sAPPL and fl-APPL are required for MTM, raising the possibility that sAPPL is a ligand of its own precursor (Figure 1). A good candidate acting downstream of APPL could be the G_o_ signaling pathway (Nishimoto et al., 1993; Okamoto et al., 1995; Ramaker et al., 2013). It remains to be determined whether sAPPL and fl-APPL are expressed by the same Kenyon cells, which would point to an autocrine mechanism, or whether fl-APPL is expressed in one specific cell type while sAPPL is secreted from another.

It has been reported that the normal physiological function of APP may be compromised by Aβ (Bignante et al., 2013). Direct interactions between Aβ fibrils and APP were described, with Aβ acting to enhance APP multimerization, a potentially toxic mechanism (Lorenzo et al., 2000; Van Nostrand et al., 2002; Lu et al., 2003; Shaked et al., 2006; Sola Vigo et al., 2009; Kedikian et al., 2010). dAβ expression enhances APPL knockdown memory impairment, raising the possibility that dAβ-induced toxicity may be caused, at least in part, by a physical dAβ/APPL interaction. Such a direct interaction could thus promote APPL cis-dimerization, a process that would compromise its function in memory (Figure 1). Furthermore, sAPPL and dAβ could have opposite functions, as sAPP was shown to disrupt APP dimers (Gralle et al., 2009). Under physiological conditions, dAβ may also interact with APPL, for example to balance and/or terminate APPL signaling. Characterization of dAβ as a neprilysin substrate would support the hypothesis of a physiological role for dAβ in memory.

It is not known whether distinct memory phases are supported by distinct APPL-mediated mechanisms. LTM is the only memory phase to depend on transcription regulation (Dubnau et al., 2003; Didelot et al., 2006). Given that APP intracellular domain (AICD), the cleavage product of APP by γ-secretase, could function as a transcription factor (Cao and Südhof, 2001; Kimberly et al., 2001; Müller et al., 2007), it would be important to know whether the APPL intracellular domain plays a specific role in LTM formation (Figure 1). It has already been reported that AICD production correlates to enhanced plasticity and memory in a TgAPP mice background (Ma et al., 2007).

The vast majority of AD cases are late-onset, happening to people at age 65 and older. Even though AD has not been described in *Drosophila*, the fly nonetheless undergoes an age-related memory impairment (AMI). This AMI exclusively concerns MTM and LTM, as none of the other memory phases decline with age (Tamura et al., 2003; Tonoki and Davis, 2012). Most strikingly, the memory phases affected by APPL knockdown in the MB are precisely those that are lost during fly aging. It is not known whether AMI could be linked to an aging-induced APPL dysfunction. It would be valuable to learn whether APPL expression and/or processing are modified during fly aging. To further explore these issues, the fly offers a suitable simplified system to decipher APP physiological function in learning and memory.

## Author Contributions

VG wrote the manuscript, TP discussed with VG and reviewed the manuscript.

## Funding

This work was supported by the Fondation pour la Recherche Médicale (DEQ20140329540).

## Conflict of Interest Statement

The authors declare that the research was conducted in the absence of any commercial or financial relationships that could be construed as a potential conflict of interest.
